# The human exposome and health in the Anthropocene

**DOI:** 10.1093/ije/dyaa231

**Published:** 2020-12-08

**Authors:** Oskar Karlsson, Joacim Rocklöv, Alizée P Lehoux, Jonas Bergquist, Anna Rutgersson, Martin J Blunt, Linda S Birnbaum

**Affiliations:** 1 Science for Life Laboratory, Department of Environmental Science, Stockholm University, Stockholm, Sweden; 2 Department of Public Health and Clinical Medicine, Section of Sustainable Health, Umeå University, Umeå, Sweden; 3 Department of Earth Sciences, Uppsala University, Uppsala, Sweden; 4 Department of Chemistry-BMC, Analytical Chemistry and Neurochemistry, Uppsala University, Uppsala, Sweden; 5 Department of Earth Science & Engineering, Imperial College London, London, UK; 6 National Institute of Environmental Health Sciences, National Toxicology Program, Durham, NC, USA

## Introduction

*‘Man is a part of nature, and his war against nature is inevitably a war against himself’* ― Rachel Carson. The term Anthropocene describes the current period, when the biophysical systems of Earth are being significantly altered by human activities. Although the great acceleration of anthropogenic influences began in the 20th century, the Industrial Revolution has often been proposed as the start of this period. Burning fossil fuels on a large scale, which was the motor of the Industrial Revolution, has disrupted the global climate, with far-reaching consequences for life on Earth. In the Intergovernmental Panel on Climate Change (IPCC) Special Report, it is stated with high confidence that human activities have significantly contributed to an increase in the global mean temperature of approximately 1°C above pre-industrial levels, and with medium confidence that trends in intensity and frequency of some climate and weather extremes have been detected, during which approximately 0.5°C of global warming occurred.^1^ The average global temperature will continue to rise if current greenhouse gas emissions continue.

The Copernicus Climate Change Service (C3S) recently reported that the 5 warmest years on record have all occurred in the past 5 years, and that 2010–19 was the warmest decade recorded. C3S also reported that 2019 was globally the second warmest year and that atmospheric carbon dioxide (CO_2_) concentrations continued to rise.^2^ During 2019 there was extraordinary ice-melt in Greenland and wildfires consumed millions of acres in Siberia, Alaska and Australia.^3^^,^^4^ Brazil’s national institute for space research (INPE) also reported fires burning at a record rate in the Amazon rainforest. The INPE reported an 80% increase in deforestation compared with that in the same period in 2018. This not only threatens local ecosystems but may affect the entire planet, because intact forest absorbs and holds large amounts of CO_2._^5^

The recent Living Planet Report by the World Wildlife Fund and Zoological Society of London provides further evidence that unsustainable human activity is threatening the planet’s natural systems.^6^ The report shows an overall decline of 60% in species’ population sizes between 1970 and 2014 because of factors such as overexploitation, habitat destruction (e.g. agricultural land expansion and logging), environmental pollution and climate change. The current species extinction rates are between 100 and 1000 times higher than previous background rate.^6^ The International Union for Conservation of Nature Red List 2019 reported that more than 27 000 species are threatened with extinction (27% of all animal, fungi and plant species assessed).^7^ A recent study of plants that have become extinct since Carl Linnaeus’ Species Plantarum has concluded that the number of known seed plant extinctions is more than four times that of the Red List.^8^

Biodiversity is a vital part of Earth’s past, present and future, and a key indicator of planetary health. The ongoing dramatic decline in biodiversity is therefore a problem, and underlying causes must be better understood for effective species conservation and human well-being. The concept of planetary health focuses on the intricate link between human health and the health of natural systems within the biosphere.^9–11^ Loss of biological diversity alters ecosystems functions and thereby reduces the ability to provide society with the goods and services, including food, necessary to flourish.^9–11^ The emergence of viruses such as the novel coronavirus (SARS-CoV-2), and global environmental change, wildlife, animal markets and food systems (which contribute largely to carbon emissions) are also interrelated.^12^^,^^13^ To increase awareness of current environmental problems and how complex interactions among climate change, infectious agents, environmental pollutants and other stressors, i.e. the exposome, may affect human health, this multidisciplinary review summarizes important scientific literature and discusses actions for a successful transformation to a safe, sustainable society.

## Climate change: impact on Earth and human health

**Figure 1:**
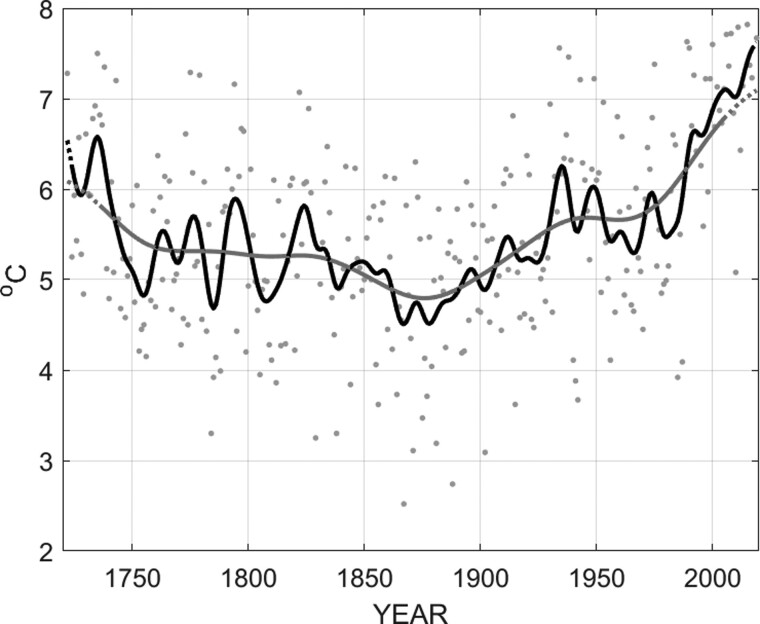
Annual mean temperature in Uppsala, Sweden, from 1722 to 2019. Annual data (black dots), 5 years running mean (black line) and 30 years running mean (grey line), dotted parts of the lines indicate fewer representative means (because of shorter time periods for the running means). Data processing according to Bergström and Moberg (2002)^15^

**Figure 2:**
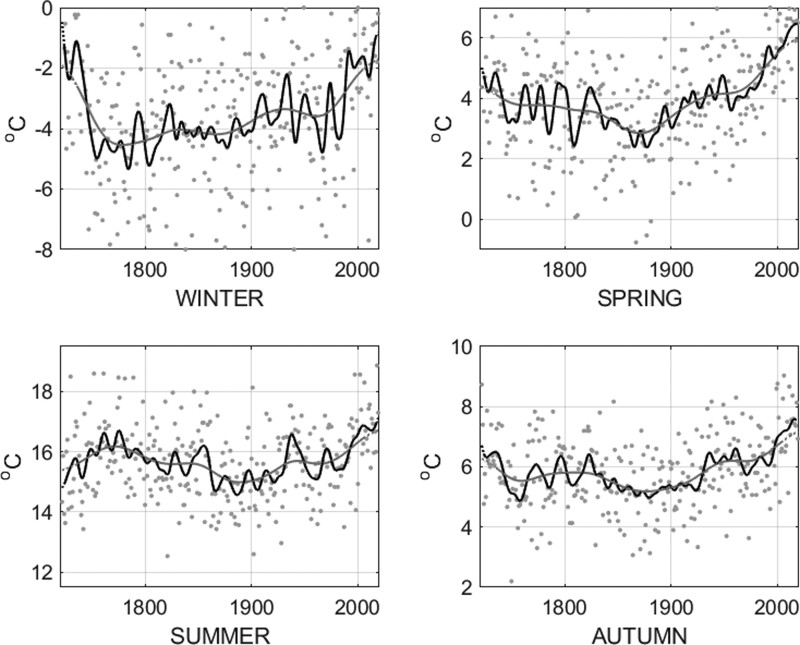
Seasonal mean temperature in Uppsala, Sweden, from 1722 to 2019. Annual data (black dots), 5 years running mean (black line) and 30 years running mean (grey line), dotted parts of the lines indicate fewer representative means (because of shorter time periods for the running means). Data processing according to Bergström and Moberg (2002)^15^

Climate is defined as the long-term average of the state of the atmosphere or the oceans; thus extended data records are necessary to establish climate change and to distinguish the differences within the natural variability of the climate system. One of the world’s longest temperature records was started in 1722 in Uppsala, Sweden, by Professor Erik Burman with support from the young Anders Celsius. Figures 1 and 2 show the annual mean temperature and seasonal mean temperatures in Uppsala from 1722 to 2019, based on this unique temperature record. The warming of the past decades is evident especially during spring; the large natural temperature variability is also observed.

The rapid growth of the human population and human activities are causing a problem: the increased pressure is destabilizing critical biophysical Earth systems and triggering environmental changes that are deleterious and catastrophic for human well-being.^16^ This is a dilemma, as the main paradigm of socioeconomic development remains largely oblivious to the risk of human-induced environmental disasters on continental to planetary scales.^16^^,^^17^ Between 1750 and 2011, cumulative anthropogenic CO_2_ emissions into the atmosphere were 2040 ± 310 GtCO_2_. The increase in CO_2_ is mainly due to burning of fossil fuels and deforestation. Approximately 40% of these emissions remain in the atmosphere; the remainder have been removed and stored on land or in the ocean, where the oceans have absorbed approximately 30% of the emitted anthropogenic CO_2_, causing ocean acidification.^4^^,^^18^

Nobel Prize Laureate Svante Arrhenius was the first to estimate the influence of atmospheric CO_2_ levels—the greenhouse effect—on Earth’s temperature, and is arguably the father of climate change science.^19^^,^^20^ Increased concentrations of CO_2_ and other greenhouse gases in the atmosphere affect the global climate, and regional climate in different parts of the world.^18^ The consequences are clearly shown for temperature, where each of the past four decades has been successively warmer at the Earth’s surface than any preceding decade since 1850. The global average combined land and ocean surface temperature data show warming of 0.85°C from 1880 to 2012. There are, however, large regional variations, with an amplification towards the Arctic areas; for example, for the Baltic Sea region, where the corresponding increase was well over 1°C.^21^

Warming induces changes in other crucial parameters such as precipitation, sea level, icemelt and snowmelt. A shift in the extent and the frequency of some types of extreme weather events has also been observed.^18^ Some areas have experienced increased frequencies of droughts and heatwaves, and other areas an increased risk of flooding. These events have consequences for forest fires, infrastructure and water availability, for example. The potential increase in extreme conditions and the possibility of ‘tipping points’, that is threshold barriers implying that it would be impossible for the considered system to restore to a healthy point, is a serious risk for society. Human activities risk triggering biosphere tipping points across a range of systems, which could induce cascade effects that further amplify ongoing environmental changes. For example, with the rapid Arctic warming, permafrost is starting to irreversibly thaw and release CO_2_ and the more potent greenhouse gas, methane (CH_4_).^22^ The subarctic boreal forest is also increasingly vulnerable; warming has triggered large-scale insect disturbances and an increase in wildfires that have led to forest dieback, potentially changing some areas from a carbon sink to a carbon source.^23^ Furthermore, Arctic warming and Greenland ice melting are drivers of an influx of freshwater into the North Atlantic, which may have contributed to the observed weakening of the Atlantic Meridional Overturning Circulation (AMOC), a system of currents central to global heat and salt transport.^4^^,^^24^ Continued melting of the Greenland ice sheet and further AMOC weakening may destabilize, for example, the West African monsoon, causing drought in Africa’s Sahel region. A slowdown in the AMOC also might dry the Amazon, disrupt the East Asian monsoon and warm the Southern Ocean, which may then increase Antarctic ice loss.^22^

Climate change affects human health too. Notably, projections have indicated that one to three billion individuals will reside outside the climate conditions that have served humanity well for the past 6000 years.^25^ As a ripple-down effect, health consequences of climate change can be direct, for example the high deaths tolls among elderly, isolated and disadvantaged populations that occur because of heatwaves. The health consequences can also be more complex, such as effects of floods beyond accidents and drownings, including water and vector-borne infectious outbreaks, displacement and socioeconomic impacts, allergic reactions to mould and increase in damp conditions, and macronutrient or micronutrient undernutrition.

The 2018 report of the *Lancet* countdown on health and climate change presents how planetary health is developing in the natural, social and human health domains.^26^ It finds that the increase in the actual average temperature that people are experiencing is greater than the global increase because temperatures are rising more rapidly in densely populated locations (e.g. large cities). In some respects, vulnerability to heat is also increasing, for instance due to the ageing of populations.^26^ Recent climate change projections indicate that worldwide, the residents of the North China Plain, one of the world’s most densely populated areas, are at greatest risk of deaths from heatwaves.^27^ The study suggests that the risk of deadly heatwaves is significantly increased because of intensive irrigation, which increases humidity and amplifies heat stress in this naturally dry region.^27^ The transmission potential of vector-borne arboviruses such as Zika, dengue, chikungunya and yellow fever also increases as a result of climate change, mainly due to enhanced virus reproduction, geographical expansion, increased abundance, higher longevity, and intensified biting behaviour of the principal mosquito vector *Aedes aegypti.*^28^ Europe has recently experienced an increase in the number of such outbreaks,^29^ probably driven by changing climate conditions and accelerating human mobility introducing and helping to spread these viruses.^30^ A similar increase has been observed for bacterial infections caused by various vibrio species that thrive in warmer waters.^26^^,^^31^

## Environmental contaminants: a threat to biodiversity and humans

Increasing societal dependence on manmade chemicals and the insufficient knowledge of their potential adverse effects is another major threat to biodiversity and human health. Understanding which environmental contaminants we are exposed to, their properties and their interactions with biological systems is therefore essential. In 1962 Rachel Carlson, an author and marine biologist from the USA, published *Silent Spring*, which is widely credited with helping to launch the modern environmental movement and raise public awareness of environmental concerns. The theme of the book is the major influences which humans have on the planet. Most of *Silent Spring* is devoted to describing the harmful effects of pesticides such as dichlorodiphenyltrichloroethane (DDT) on ecosystems; cases of human pesticide poisoning, cancer and other illnesses attributed to pesticides are also discussed.^32^ The increased public concern over the environment, human health and human safety spurred the establishment of several important regulatory agencies and non-governmental organizations (NGOs) that focus on these areas. Since the publication of this seminal book, society has also had an improved understanding of how environmental contaminants affect biological processes, and agencies have used risk assessment based on toxicological data to reduce harm, by imposing regulations and public health policies.

Despite these important advances, some of the environmental lessons learned appear to need re-teaching. The latest data from two research programmes, led by the French National Centre for Scientific Research and France's National Museum of Natural History in Paris, show that in less than two decades, one-third of all birds have disappeared from the French countryside. This suggests that the silent spring predicted by Rachel Carson may yet arrive. Some species, such as the meadow pipit (*Anthus pratensis*), are more severely affected and have declined by almost 70%. Widespread use of pesticides and more intensive land use by farmers are suspected to be the main factors causing the observed decline. Pesticides can reduce insect populations to a level that makes it difficult for birds to find food. Accordingly, an 80% decline in flying insect biomass has been reported, and a loss of more than 420 million birds, over 30 years in Europe.^33^^,^^34^ A recent review of 73 historical reports confirms a dramatic insect decline and estimates that over 40% of the world’s insect species are threatened with extinction.^35^ This widespread loss includes pollinating insects critical for the ecosystem and agricultural productivity across the world.^36^ In addition to pesticide use and habitat destruction, farming is also a source of aquatic and terrestrial pollution through nitrogen and phosphorus loading from fertilizer and manure applications, which is another major cause of biodiversity loss and ecosystem dysfunction.^37^^,^^38^

Plastic pollution is another growing global problem. Due to increasing plastic production and poor recycling rates, most plastic is released into the environment. Large pieces of plastic eventually break down into smaller-sized pollutants, called microplastics when they are less than 5 mm in length. Microplastics can further divide into smaller-sized nanoplastics (<100 nm), which are equally toxic but more difficult to detect in the environment and more mobile.^39–41^ Microplastics and nanoplastics are also intentionally produced by various industries. All plastic pollutants have associated toxic effects on the biosphere in terrestrial and aquatic environments.^39–41^ Therefore, more research is necessary to elucidate the impact of this type of pollution on biota and human health and to aid future risk management.^40^^,^^42^

The pulp and paper industry was one of the most polluting industries in the world, discharging gas, liquid and solid wastes into the environment, until the implementation of strict regulations.^43^ However, legacy-contaminated sediments can still be found at many sites, forming large fibrous deposits called fibrebanks on the bottom of water bodies.^41^^,^^44^ Depending on the industrial process used by each factory and the pesticides used during tree growth, the resulting fibrebanks have different physical structures and concentrations of persistent organic pollutants (POPs) such as DDT, polychlorinated biphenyls (PCBs), hexachlorobenzene (HCB) and metallic contaminants (Cd, Co, Cr, Cu, Hg, Ni, Pb, Zn, As). Many of these pollutants have been found at concentrations representing a potential threat to the environment.^44–46^ The organic nature of these sediments also creates an anoxic environment and releases greenhouse gases (CH_4_ and CO_2_). Only a few sites in the world have been remediated, and remediation options are currently under investigation to efficiently limit the dispersal of contaminants and prevent bioaccumulation.^47^

In addition to the adverse effects on biota and ecosystems, environmental contaminants are a direct threat to human health. Exposure to air pollution such as tropospheric ozone and particulate matter (PM), or the hundreds of thousands of manmade chemicals that contaminate our environment, are major risk factors for many human chronic diseases. A recent comprehensive study estimates that diseases caused by pollution were globally responsible for 9 million premature deaths in 2015 (16% of all deaths).^48^ The deaths attributed to pollution are three times higher than from AIDS, tuberculosis and malaria combined.^48^ In the most severely affected nations, pollution-related diseases are estimated to be responsible for more than 25% of all deaths. In all countries, disease caused by pollution is most prevalent among minority and marginalized populations.^48^ The study further estimates that air pollution causes 6.5 million of the pollution-related premature deaths, which is in line with the updated World Health Organization estimate of 7 million deaths per year caused by ambient (outdoor) and household air pollution.^48^ However, the true contribution of pollution to the global disease burden is probably underestimated because the adverse effects of many environmental contaminants are poorly understood. The vast majority of environmental contaminants have not been thoroughly tested for their toxicity and safety for environmental or human health. Characterization of contaminant exposures and possible adverse effects of chemical pollutants such as pharmaceuticals, pesticides, nanomaterials, and endocrine disruptors is therefore essential.

The adverse effects of a contaminant do not only depend on the dose and its properties; the timing of the exposure is also crucial. A growing amount of data demonstrates that children are at high risk of pollution-related disease. Exposures to even low contaminant levels during vulnerable time periods *in utero* and in early infancy can disturb development and lead to disease in childhood or even later in life.^49^^,^^50^ There has been an increasing interest in whether environmental factors modulate the establishment and maintenance of epigenetic modifications and thereby affect gene expression and phenotype in humans and animals.^51–54^ Epigenetic mechanisms such as DNA methylation, histone modifications and non-coding RNA are important pathways for long-term effects of developmental exposure.^49^^,^^55^ They may also mediate multigenerational effects of environmental exposure through epigenetic inheritance.^56–58^

There is a need to move toxicology, from hazard and risk assessments built on characteristic morphological endpoints in animal studies, towards a mechanism-driven integrated approach that includes computational modelling and molecular, and *in vitro* studies. Recent technological developments, such as omics and imaging techniques, the ability to generate human-induced pluripotent stem cells (iPSCs), and gene editing by CRISPR/Cas9, are examples of advances that allow the generation of novel high-content data, providing the potential to accelerate the discovery of toxicant pathways, modes of action and specific chemical targets in a systems toxicology approach.

Several US federal agencies are cooperating to advance the state of toxicity testing. Toxicology in the 21st century (Tox21) is a programme integrating rapid *in vitro* testing approaches among the National Institute of Health, specifically, NIEHS and NCATS, the US Environmental Protection Agency (EPA), and the US Food and Drug Administration. ToxCast is the rapid screening programme of the US EPA and has involved testing of several thousand compounds in over 800 *in vitro* assays. Tox21 has tested nearly 10 000 compounds in a more limited test battery of approximately 70 assays focused on receptor binding and oxidative stress assays. More recently, there has been a focus on developing alternative *in vivo* animal models such as using zebrafish and *C. elegans* for toxicity testing. Application of advanced computational approaches has helped define the mechanisms and modes of action of chemicals, leading to the ability to predict whether the effects observed in experimental models also occur in humans. In this context, the adverse outcome pathway (AOP) framework has started to gain traction, because it offers a model for capturing available knowledge describing the linkage between mechanistic data and the toxicity endpoints required for regulatory assessments.^59^

## The exposome: contaminants, climate, microbiota and other stressors interact

Historical discussions of nature (genome and inherited characteristics) and nurture (life influences) have directed the understanding of biology and disease. The most recent research has had a bias towards searches for genetic explanations of disease while ignoring the contribution of pollution. However, after thousands of genome-wide association studies (GWAS), a relatively small proportion of chronic disease can be attributed to genetic factors alone. Even in developed countries where pollution generally is lower than in developing countries, most chronic diseases probably result from the combination of genetics and environmental exposure. A recent analysis of 28 chronic diseases in monozygotic twins revealed that the proportions of disease risks attributable to genetics ranged from 3% to 49% with a median of only 19%.^60^ Twin studies further suggest that cancer risks attributable to genetic factors may be approximately 10%.^61^ Results from over 400 GWAS indicate that the heritability of degenerative diseases is also typically around 10%.^62^^,^^63^ Hence, environmental factors are well-established major risk factors for many diseases, and the necessity for more research and better tools to determine environmental contributions is vital.

**Figure 3:**
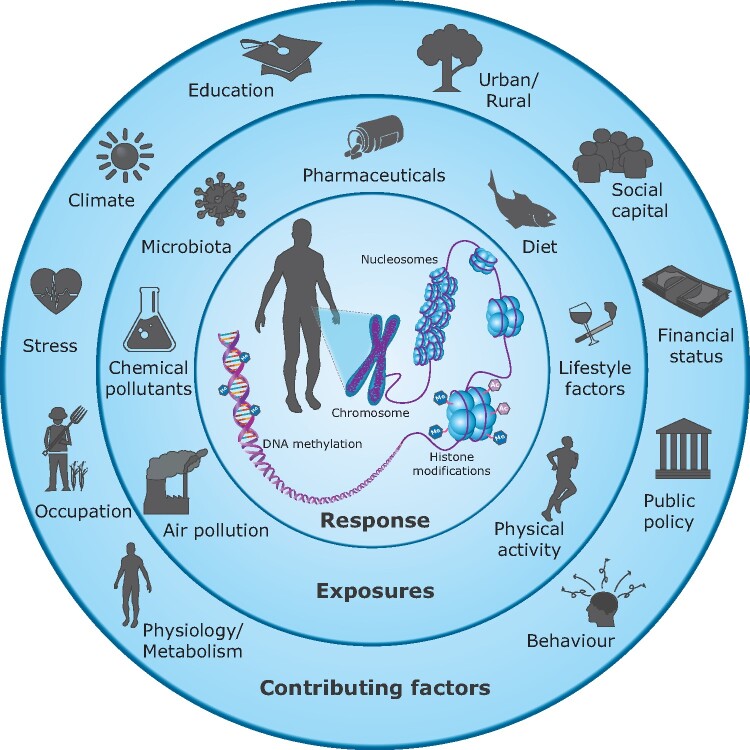
The human exposome. Environmental exposures are major risk factors for human diseases, and only a small proportion of chronic illness can be attributed to genetic factors alone. Systematic information on the human exposome—the lifetime sum of environmental exposures—is therefore important to reduce the overall burden of disease. Humans are continuously exposed to many contaminants and other stressors that can induce adverse effects, independently or when they interact. For example, the consequences of climate change include adverse health effects through heatwaves and increased vector-borne infectious outbreaks and the potential to alter environmental concentrations and biological effects of pollutants. The individual response to current exposures and susceptibility to disease are influenced by genetics, epigenetics, physiology and health status, which involve changes in biological pathways caused by earlier exposures. Epigenetic mechanisms such as DNA methylation, histone modifications and non-coding RNA are important pathways for effects of the exposome. The exposures can alter epigenetic modifications and thereby turn on, or off, genes. Investigating the effects on epigenetic processes by using omics tools can therefore provide evidence of responses to a set of exposures and improve the understanding of human disease

The exposome paradigm was first proposed in 2005 to raise awareness of the importance of comprehensive exposure assessment in human health research, as a complement to the large investment in genetic research.^64^ The exposome can be defined as an individual’s cumulative lifetime environmental exposure and related biological responses. Under the exposome concept, all non-genetic factors contributing to disease are environmental, including air pollutants, chemicals, drugs, radiation, microbiota, climate and psychosocial stress (Figure 3).^65–67^ The exposome can alter important biological pathways through, for example, epigenetic mechanisms such as DNA methylation, histone modifications and non-coding RNA, which alter the expression of key genes.

Today, rapid advances in omics techniques such as epigenomics, transcriptomics, proteomics, metabolomics and metagenomics and systems biology provide tools to gain unprecedented insight into human disease. In combination with genome data, systematic information on the human exposome could significantly improve understanding of human disease. Traditionally, environmental exposures are measured through questionnaires, geographical information or targeted analytical methods.^68^ The development of high-resolution mass spectrometry and approaches that combine targeted chemical analysis with suspect-screening and non-target analysis has opened new research opportunities by allowing the screening of many chemicals simultaneously (i.e. the chemical exposome).

The exposomic paradigm represents a shift from a targeted hypothesis-driven model to a broader, but complementary, discovery-based approach that can lead to a better understanding of current environmental exposures and their impact on biota and human health.^65^^,^^66^^,^^69^ A more comprehensive analysis of chemical contaminants allows the discovery of previously unidentified and emerging exposures, with unknown impacts on biological systems. For example, although several halogenated contaminants have been thoroughly studied in polar bears (*Ursus maritimus*) since the 1970s,^70^ a recent non-targeted mass spectrometry study discovered hundreds of new contaminants, including novel PCB metabolites and many chlorinated or fluorinated compounds not previously detected, illustrating the strength of this analytical strategy.^71^ Furthermore, by examining the toxicological impact of complex real-world chemical mixtures—instead of one chemical at a time—it may be possible to more accurately identify risk factors and underlying mechanisms.^72^ This more advanced type of research is critical because chemicals in mixtures can interact and may have greater, or lesser, than additive effects on biological processes.^73^

Equally important is studying interactions with other parts of the exposome by considering all types of chemical, physical and biological stressors and query associations with particular outcomes or biomarkers. For example, a growing number of studies have reported the important role of the gut microbiome in human health and disease. It has also been shown that pharmaceuticals can affect the gut microbiome and vice versa, but the complex relationship and its possible role in the aetiology of disease and response to medical treatment warrant further research.^74^^,^^75^ A better understanding of the bidirectional interactions between microbes and pharmaceuticals may result in new therapies or improvements to how medicines are prescribed.

In addition, closely linked factors such as diet and environmental contaminant exposures can strongly influence gut flora.^76^^,^^77^ A recent study found that people immigrating from Thailand to the USA demonstrated a clear Westernization of their gut microbiome, accompanied by an increased risk of obesity, illustrating how different parts of the exposome may interact and affect health.^76^ Pathogens and environmental pollution can also interact and worsen adverse health effects. For example, residents of regions with high air pollution are more likely to have compromised respiratory and cardiac systems and may therefore be more vulnerable to infectious agents targeting the respiratory system. During the severe acute respiratory syndrome (SARS) outbreak in China, a study demonstrated that SARS patients were twice as likely to die from the disease when they lived in areas of high air pollution.^78^ Subsequent studies have shown similar associations between air pollution exposure and mortality rate in the ongoing coronavirus pandemic (COVID-19).^79^^,^^80^ Data also suggest that air pollution in the form of PM has the potential to enhance the transmission by acting as carriers of exhaled virus droplets, which in conditions of clean air and atmospheric turbulence quickly evaporate and/or disperse.^81^

It is important to understand that the human exposome is closely interconnected to planetary health. One of the consequences of global warming that has recently attracted attention is the potential to alter the environmental distribution and biological effects of contaminants. Climate change-induced alterations in food webs, lipid dynamics, icemelt, snowmelt and organic carbon cycling could increase levels of POPs in water, soil, and biota.^82^ For example, a study predicts an over 50% increase in tissue methyl mercury concentrations in Atlantic bluefin tuna (*Thunnus thynnus*), frequently consumed by humans, because of the recent increase in seawater temperature, which is consistent with measured levels from 2017.^83^ Regional precipitation patterns are also predicted to be affected by climate change. Areas subject to reduced precipitation may experience enhanced air pollution including the volatilization of environmental contaminants into the atmosphere. Regions with increased precipitation will have lower levels of air pollution but may instead experience enhanced surface deposition of airborne POPs and increased run-off of pesticides.^82^ Hence, populations could experience altered exposure to contaminants that depends on their region and diet.

Climate change may not only affect the composition of an individual’s exposome but could also enhance the toxicity of the contaminants. For example, the human health impacts of ozone and PM will be exacerbated with the increasing temperatures.^82^^,^^84–86^ A recent study of heatwave mortality in Chinese cities shows that regions with higher PM_2.5_ levels have an increased vulnerability to heatwaves.^87^ Other potential interactions between climate change and pollutant exposure include increased vulnerability to pathogens, through toxicant-induced immune system impairments and climate shifts in disease vector range.^88–90^ Experimental studies have also shown that global warming could be particularly harmful to pollutant-exposed wildlife.^91^^,^^92^ The complex interactions between climate change and environmental contaminants may be especially challenging for species living at the border of their physiological tolerance limit where acclimation capacity is low.^82^

## Responding to the global environmental change

The urgency of the current situation is due to the alarming predicted scientifically-based consequences and has inspired the younger generation. For instance, school strikes in support of the environment are held frequently worldwide, prompted by the young Swedish student Greta Thunberg’s weekly protests since August 2018. NGOs and activists are important for spreading awareness and knowledge among society and capturing the attention of the world’s leaders, and eventually acting as a catalyst for necessary change.

Despite the importance of such actions, an individual’s power remains limited by the choices offered by each nation. Therefore, the implementation of strong regulatory frameworks to protect the environment is essential. Several national and international regulations and agreements have been implemented to improve environmental quality. In 2015, the United Nations (UN) agreed on new goals for sustainable development within Agenda 2030, covering economic, social and environmental dimensions. The Paris Agreement is another response milestone that builds on the UN Convention and unites countries in a common cause to undertake ambitious efforts to mitigate climate change and adapt to its effects.

By maintaining the global temperature rise this century to well under 2°C above pre-industrial levels and pursuing efforts to limit the temperature increase even further to 1.5°C, the ecological and human health impacts and the risk of tipping points are substantially lowered. This requires appropriate financial investments, a new technology framework and enhanced capacity-building for sustainable development that also supports the actions of developing countries. However, with current levels of fossil-fuel burning, even a very sharp decrease in emissions would be unlikely to limit temperate increases to 1.5°C, or even 2°C. Most assessments have indicated the need for negative emissions, where global-scale implementation of geological carbon capture and storage (CCS) is one of the major concepts.^93^^,^^94^ CCS involves the capture of CO_2_ from large point sources, such as fossil-fuel-burning power stations, refineries, cement works, aluminium smelters and other industrial plants, followed by transport and injection deep underground, into porous rock containing salty water or depleted hydrocarbon reservoirs.^1^^,^^95^ The major costs are associated with the separation of CO_2_ from the exhaust stream of industrial processes (capture), and most public concern focuses on the safety of CO_2_ long-term storage.^95^ Recent research into the flow processes that occur underground has demonstrated that safe storage is possible—as demonstrated already in several sites worldwide—when the pressure increases in the subsurface are limited.^96^ Over time, the CO_2_ becomes less mobile, as it dissolves in water, and sinks, or becomes trapped as pore-space bubbles, which increases storage security.^96^^,^^97^ The study of natural analogues suggests that it is highly unlikely that any significant fraction of the CO_2_ will escape on a 10 000-year-timescale.^98^ Furthermore, the hazards associated with leaks, principally asphyxiation, are minimal, based on the analysis of sites in Italy where the CO_2_ of volcanic origin escapes to the surface.^99^

A recent assessment demonstrated how CCS can be implemented to decarbonzse electricity production and heavy industry in the UK.^94^ Worldwide adoption of CCS would create an activity—in terms of volumes of fluids injected—similar in size to the oil and gas industry that should eventually be replaced by renewable energy technologies and sources. If biofuel is burned alone or in combination with, for instance, natural gas, the process can become carbon negative, in that overall CO_2_ is extracted from the atmosphere on a lifecycle basis.^97^ Other, more speculative, ideas focus on the capture of CO_2_ directly from the atmosphere, either for injection underground or with a conversion to a solid form.^100–102^

## Concluding remarks

Many of the names Carl Linnaeus gave individual species in the 18th century continue to be used, including the name for humans, *Homo sapiens*, the rational human. Today, three centuries later, society must make rational choices urgently to mitigate and adapt to climate change, reduce environmental contamination and reverse the devastating trend of biodiversity loss. Scientific evidence has repeatedly demonstrated that unsustainable human activity is threatening the natural life-supporting systems on Earth.

This review illuminates the importance of cross-disciplinary work to better understand and solve some of the complex challenges of humanity in the Anthropocene. The increasing greenhouse gas emissions are the main cause of global climate change, and the consequences are not limited to temperature changes. Climate change is also contributing to the dramatic decline in biodiversity and to adverse human health effects because of, for example, heatwaves, increased vector-borne infectious outbreaks and potentially increased toxicity of environmental pollutants. Although the potential impact of humans’ past and present activities is now better understood, this knowledge must be improved, including the understanding of Earth’s systems and the effects of the human exposome on the global disease burden, where epidemiologists and biostatisticians play important roles.

There has been a clear focus on finding the genetic underpinnings of disease, but it is now evident that environmental exposures are major risk factors for most diseases and that a relatively small proportion of chronic illness can be attributed to genetic factors alone. Therefore, it is important to: focus on the environment; leverage new omics methods and other technology developments to characterize the exposome in detail; better understand the interactions between its different components and the genome; and determine the impact on human health in large epidemiological studies. This focus includes optimal use of resources such as cohort studies and national registers, as well as building new exposome-focused cohort studies. These types of complex studies will be challenging and require cross-disciplinary collaboration. Although several statistical approaches, most adapted from disciplines such as genetics and epigenetics, have been proposed to estimate the effects of multiple exposures,^103^ further development of suitable data analysis methods is necessary. The individual response to current exposures, and susceptibility to disease, is influenced by genetics, epigenetics, physiology and health status, which involves changes in biological pathways caused by earlier exposures. Thus, it is important to conduct molecular epidemiological studies that include vulnerable developmental time periods *in utero* and in early infancy, which can lead to disease in childhood and later in life.^49^ Although studies have demonstrated that interactions between global warming and contaminant exposures can lead to serious health consequences, further research on the human health effects of pollutants under projected climate change scenarios is necessary.

This review has also illustrated that human and planetary health are interconnected. Therefore, society must act accordingly. Despite several national and international regulations and agreements, emissions continue to increase, showing that stronger governmental efforts are necessary worldwide. Refining food production, better use of resources, less waste, improved recycling, lower CO_2_ consumption, implementation of CCS and sustainable solutions for a fossil-free future are some of the priorities. In addition, improved toxicity testing, global chemical regulation and green chemistry, to produce safer and more environmentally friendly chemicals and materials, are important. Given the major role of economic activity in the ongoing anthropogenic changes, a more rapid integration of economics into the core of sustainable development, and vice versa, is necessary.^14^

The relation between the environment and economy is diverse. The environment provides vital natural resources and acts as a sink for emissions and waste. Unsustainable production and consumption result in pollution and other pressures on the environment. Degraded environmental quality subsequently affects economic growth and human well-being, for example by reducing the quantity and quality of natural resources or by causing adverse health impacts, including death. The economic system largely builds on linear principles focused on throughput, optimization and cost-benefit efficiency, and was developed when the population was lower than it is today. However, on a finite planet such as Earth, society must carefully define and remain within planetary boundaries for sustainable global economic development. This requires competent political leadership and ambitious environmental policies, because the markets cannot correct these problems alone. A long-term vision that involves attainable but impactful targets and effective instruments and metrics for successful implementation is therefore necessary in political goals and actions. Key tools such as ‘ecosystem services’ should be further explored for improved communication between researchers and decision makers, giving nature a distinct economic value and improving the consideration of environmental aspects in societal decision making.

The ongoing COVID-19 pandemic is a global disaster. Under measures to increase social distancing and prevent the spread of the coronavirus, factories and businesses have closed worldwide, and social movement and travel have been restricted, resulting in an economic slowdown but also temporary improvements in air quality in some regions. However, because several national governments are repealing standing emissions regulations in an attempt to financially support industry, the response to this pandemic threatens to worsen the long-term health impact of air pollution, which kills millions of people per year^48^ and might, as described above, enhance transmission of the coronavirus^81^ and increase the risk of death from COVID-19.^79^^,^^80^ Hence, as world leaders respond to COVID-19 with upcoming major economic stimulus investments, it will be essential to simultaneously minimize anthropogenic changes. Addressing environmental and climate change in the recovery from COVID-19 would probably help reduce the risks of emerging infectious disease and other public health problems in the near and distant future. These benefits must affect the entirety of society and thus create win-win situations where human health and the environment are advanced in a sustainable manner. A combination of individuals’ efforts, political power and scientific knowledge and development is the key to improving the health of the population and planet.
